# A case report and mechanism analysis of a normal phenotype mosaic 47, XXY complicated by paternal iUPD (9) who had a normal PGD result

**DOI:** 10.1186/s12881-019-0897-5

**Published:** 2019-11-07

**Authors:** Dan Li, Yun Wang, Nan Zhao, Liang Chang, Ping Liu, Chan Tian, Jie Qiao

**Affiliations:** 1 Peking University Third Hospital, 49 North Garden Road, Haidian District, Beijing, 100191 People’s Republic of China; 2 National Clinical Research Center for Obstetrics and Gynecology, Beijing, 100191 China; 3 Key Laboratory of Assisted Reproduction (Peking University), Ministry of Education, Beijing, 100191 China; 4 Beijing Key Laboratory of Reproductive Endocrinology and Assisted Reproductive Technology, Beijing, 100191 China; 5 Beijing Advanced Innovation Center for Genomic, Beijing, 100871 China; 6 Peking-Tsinghua Center for Life Sciences, Peking University, Beijing, 100871 China

**Keywords:** Parental iUPD (9), Mosaic Klinefelter syndrome, Preimplantation genetic testing for chromosomal structural rearrengements (PGT-SR), Prenatal diagnosis

## Abstract

**Background:**

Uniparental disomy (UPD) refers to the situation in which two copies of homologous chromosomes or part of a chromosome originate from the one parent and no copy is supplied by the other parent.

**Case presentation:**

Here, we reported a woman whose karyotype was 46, XX, t (1;17)(q42;q21), has obtained 5 embryos by intracytoplasmic sperm injection (ICSI) after one cycle of in vitro fertility (IVF). After microarray-based comparative genomic hybridization (array-CGH) for preimplantation genetic testing for chromosomal structural rearrangements (PGT-SR), two embryos were balanced, one balanced embryo was implanted and the patient successfully achieved pregnancy. Amniocentesis was performed at the 19th week of gestation for karyotype analysis and single nucleotide polymorphism (SNP)-array test. The result of karyotype analysis was: mos 47, XXY [19]/46, XY [81]; SNP-array results revealed 46, XY, iUPD (9) pat. After full genetic counseling for mosaic Klinefelter’s syndrome and paternal iUPD (9), the couple decided to continue pregnancy, and the patient gave birth to a healthy boy. The newborn is now 3.5 years old, and developed normally. This case will provide counseling evidences of paternal iUPD (9) for doctors.

**Conclusions:**

This is the first case report of paternal iUPD9 with mosaic Klinefelter’s syndrome, and no abnormality has been observed during the 3.5-year follow-up. Further observation is required to determine whether the imprinted genes on the chromosomes are pathogenic and whether recessive pathogenetic genes are activated.

## Background

Uniparental disomy (UPD) refers to the situation in which two copies of homologous chromosomes or part of a chromosome originate from the one parent and no copy is supplied by the other parent [1]. The frequency of constitutional UPD cases has not yet been exactly determined in the general human population. The incidence of UPD in newborns is approximately 1/3500 [1]. UPD may cause diseases secondary to the activation of imprinted genes or due to inheritance of recessive pathogenic genes. At present, identified chromosomal fragments bearing imprinted genes include 6q24, 7p11.2-p12, 7p32.2, 11p15.5, 14q32.2, 15q11-q13 and 20q13.3 [2]. In addition, newly identified imprinted genes can be found in the imprinted gene database (http://www.geneimprint.com/). More than 100 imprinted genes have been reported. At present, there are more than 2800 UPD cases on record [3]; however, very few paternal UPD (9) cases have been reported. There has been no case report of normal chromosome 9 with homozygous paternal UPD9; therefore, it is unknown whether paternal iUPD9 (isodisomy) is pathogenic. In this study, we report a patient with a balanced translocation of chromosomes 1 and 17 who underwent preimplantation genetic testing for chromosomal structural rearrangements (PGT-SR) during gestation period that revealed a fetal karyotype of 47, XXY mosaicism complicated with iUPD (9) pat. The newborn was followed until age 3.5 years old and developed normally.

## Case presentation

**Fig. 1:**
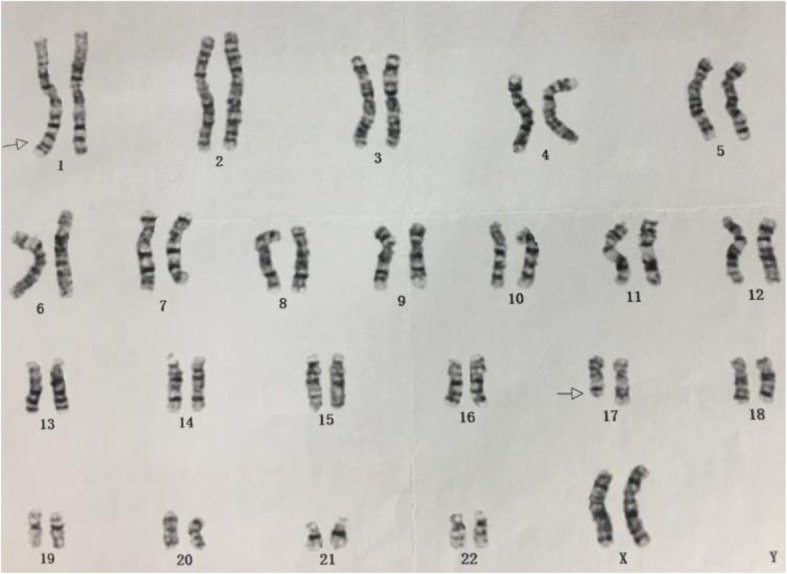
Metaphase spread in G band Trypsin Giemsa (GTG)-banding obtained from the mother’s blood lymphocytes showing 46, XX, t (1;17)(q42;q21). Arrows show abnormal chromosomes

The woman have had two fetuses with congenital heart disease and terminated pregnancies at 29 and 31 years old in 2012 and 2014, respectively. The karyotype of the woman was 46, XX, t (1;17)(q42;q21) (Fig. 1), and that of her husband was 46, XY.

**Fig. 2:**
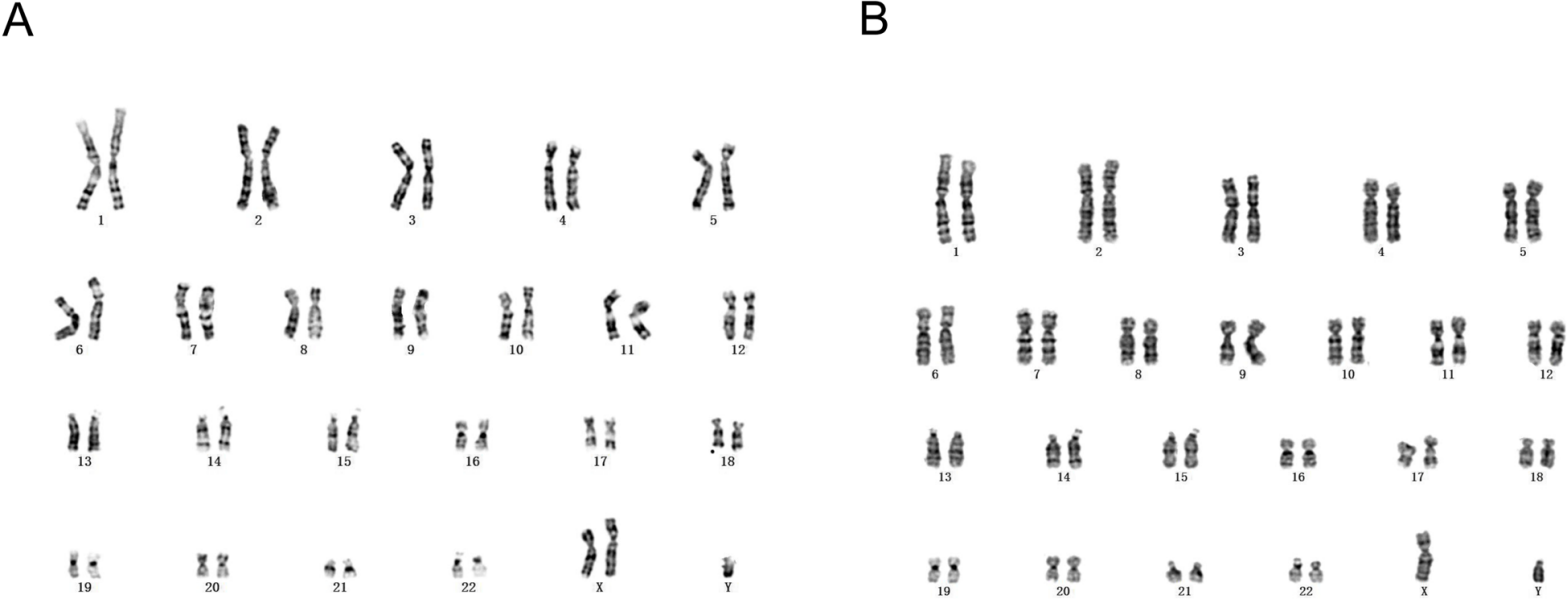
Metaphase spread in GTG-banding obtained from the amniocentesis at the 19th week of gestation. The result is 47, XXY [19] (**a**)/ 46, XY [81](**b**)

**Fig. 3:**
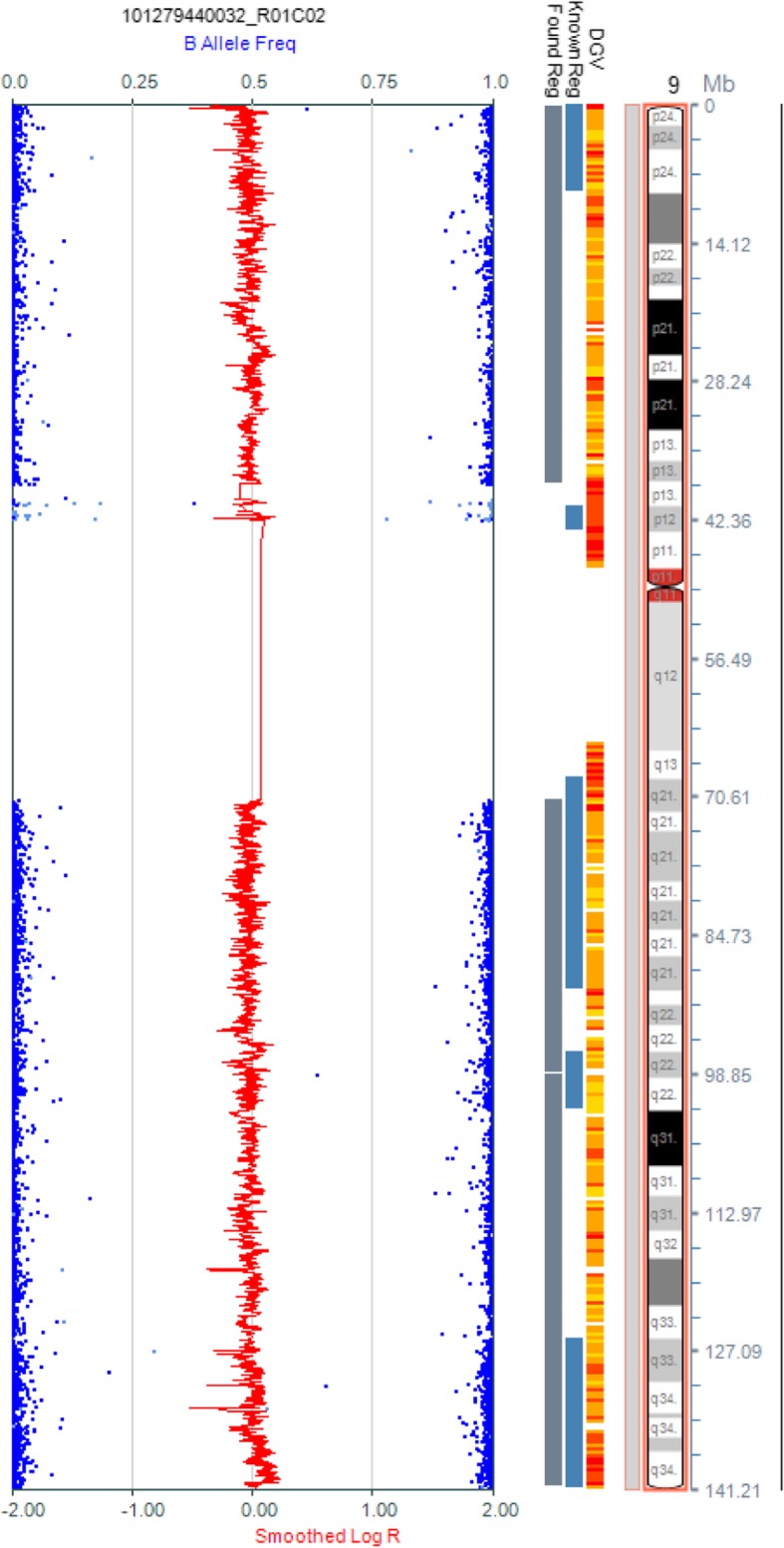
The SNP-array results of the amniocentesis revealed 46, XY, iUPD (9)

In 2015, the patient underwent in vitro fertilization (IVF) followed by PGT-SR to assist in pregnancy. Eighteen eggs were acquired, and 5 were fertilized by intracytoplasmic sperm injection (ICSI). Blastocyst biopsy was performed on day 6 embryos for microarray-based comparative genomic hybridization (array-CGH) for PGT-SR. Two embryos were balanced, and 3 embryos were unbalanced (Table 1). One balanced embryo was implanted during the thawing cycle, and the patient successfully achieved pregnancy. Amniocentesis was performed at the 19th week of gestation for karyotype analysis. To detect whether there were microdeletion/microduplication that can’t be detected by PGT-SR, single nucleotide polymorphism (SNP)-array was undertaken [4]. The result of karyotype analysis was as follows: mos 47, XXY [19]/46, XY [81] (Fig. 2); SNP-array results revealed 46, XY, iUPD (9) (Fig. 3). Peripheral blood from both parents was extracted for SNP-array analysis, and SNP loci analysis and comparison were performed then. Fetal UPD9 was identified to be of paternal origin. To further determine the fetal mosaicism, cord blood puncture was performed at the 26th week of gestation. The karyotype was mos 47, XXY [17]/46, XY [83], and SNP-array results were the same as in the previous test. After full genetic counseling, the couple decided to continue pregnancy. The patient gave birth to a healthy boy by cesarean section at 38 weeks, with a body length of 49 cm and a weight of 3250 g. Apgar score was 10. Phenylketonuria (PKU) and thyroid function screening of the neonate were normal. The newborn is now 3.5 years old and developed normally.

**Table 1:** Result of PGD

aCGH result	Number of embryos	Result
+ 16, -22	1	Give up
+3p	1	Give up
+ 2,-6,+ 15	1	Give up
Balanced	2	Transplanted

## Discussion and conclusion

To date, there has been no case report of chromosome 9 disomy with complete paternal homologous UPD9 and sex chromosomal abnormality. We discovered a 47, XXY/46, XY, iUPD (9) pat fetus in the second-trimester of pregnancy. At present, the child is 3.5 years old, growth and development are normal. The biggest difficulty for us is to judge the prognosis before delivery. In addition, we also analyzed the possible reason and mechanism so that can raise the advice to subsequent clinical practice.

According to the current cognition, the adverse consequences of UPD may include imprinted gene diseases, activation of recessive pathogenic genes or genetic effects caused by partial chromosome imbalance. To define if there were paternal imprinted genes on chromosome 9, we searched publications and database. Based on reports by *Uniparental Disomy (UPD) in Clinical Genetic*s [2], there is no case report of paternally imprinted genes or activation of recessive pathogenic genes on chromosome 9. However, by searching the database of imprinted genes (http://www.geneimprint.com/), we found that GLIS family zinc finger 3 (GLIS-3) is an imprinted gene of paternal expression located on 9p24.2. GLIS-3 is a member of the zinc finger family and a member of the transcription factor superfamily, serving as an important gene in many physiological functions and the growth process of the fetus. It is both a transcription activator and a suppressor and is involved in the development of pancreatic β cells, thyroid, eye, liver, and kidney. Double allelic mutations of this gene are associated with neonatal diabetes and congenital hypothyroidism [5]. For our case, during the prenatal phase, fetal samples were sequenced by commercial companies according to the couple’s personal choice, but no known pathogenic mutations were identified (results not shown). This suggests that the child is at risk of neonatal diabetes and congenital hypothyroidism because of the probable imprinting gene GLIS-3.

By now, only seven UPD9 cases are reported. Kaiser-Rogers et al. [6] first reported a case of androgynous (dizygotic female) twins of paternal UPD 9 in which intrauterine growth retardation was identified in prenatal diagnosis; the fetuses subsequently died in utero. Yang et al. [7] reported a 20-year-old female with paternal homodisomy UPD9 who had juvenile amyotrophic lateral sclerosis type 16 due to frameshift mutation of the Sigma non-opioid intracellular receptor 1 (SIGMAR1) gene on 9p13.3. Chen et al. [8] reported a phenotypically normal child with a karyotype of 47, XY, +mar [25]/48, XY, +mar, +r (9) [4]/47, XY, +r (9) [1]/46, XY [6], of which the circular chromosome 9 and the marker chromosomes were both paternal. Although the patient’s phenotype was normal, the child’s genome was unbalanced, containing a partial duplication of 9p13.1-p22.3. Both the two reported paternal trisomy 9 mosaicism cases [9, 10] demonstrated developmental delay. One case exhibited intrauterine growth retardation, and the other case exhibited severe developmental retardation, congenital cerebral dysplasia, and congenital heart disease at 22 months. Carvalho et al. [11] reported a case of paternal UPD9 with 9q12-q21.11 duplication. Our case is the seventh.

In addition, the fetus also exhibited 47, XXY/46, XY mosaicism. To eliminate false mosaicism in amniotic fluid culture, we performed umbilical cord blood puncture at the 26th week of gestation. The karyotype and mosaicism ratio were similar to those of the amniotic fluid culture, indicating that mosaicism did exist in the fetus. The XXY chimeric karyotype is likely to cause oligospermia or infertility in adulthood, but the extent of this phenotype depends on the mosaicism in the gonad, which is difficult to estimate due to the difficulty of sampling. Aneuploidy analysis of two males of 47, XXY/46, XY (XXY mosaicism of 70 and 78%) by Morel et al. [12] indicated that the chimeric male had oligospermia, but more than 90% of the sperm were haploid, and the production of 24 XY and 24 XX gametes was higher compared with the normal control group. The higher the XXY mosaicism ratio is, the higher the probability of producing nonhaploid sperms. In this case, the XXY mosaicism ratio of the fetus is 19%. Therefore, oligospermia may happen at the reproductive stage, but PGT-SR-assisted pregnancy may not be necessary. Moreover, careful ultrasound examinations during pregnancy revealed no fetal organ and facial abnormalities or intrauterine stunting. After genetic counseling, the couple decided to continue pregnancy.

The result of the PGT-SR for the fetus was normal but it was not in accordance with that of prenatal diagnosis. Possible reasons for this discrepancy are as follows. First, we used the array-comparative genomic hybridization (array-CGH) method in PGT-SR, which cannot detect UPD. The SNP-array method was used in prenatal diagnosis and produced results that differed from PGT-SR. This leads us to consider whether we need to check UPD during the PGT-SR phase. It has been reported [13] that the probability of UPD in embryos is 0.06%; therefore, there is no need of routine UPD detection. Our results also support this conclusion. Second, because the chimeric ratio of this case was only 20%, the mosaicism eluded detection due to the limited detection sensitivity of the array-CGH gene chip. Third, although blastocyst biopsy is currently recognized as a good method for detecting embryonic mosaicism, studies on embryonic mosaicism have demonstrated [14–16] that the inconsistency rate between tested trophectoderm biopsy and the inner cell mass is approximately 3 to 4%. In addition, studies have indicated [17] that if chimeric cells are evenly distributed across the blastocyst, biopsy of 27 cells is required to represent the entire blastocyst. However, our biopsy only took a few cells from one place in the blastocyst trophoblast, much less than 27 cells. Moreover, chimeric embryos may not be detected since mosaicism is often uneven, or the inner cell mass and trophoblast may both exhibit heterogeneous mosaicism.

According to the literature, paternal UPD is primarily iUPD, which is the inheritance of two copies of one parental chromosome, is usually due to monosomic rescue [2]. Therefore, it is speculated that fetal iUPD9 was due to a maternal meiosis II error, resulting in fertilization of an egg without chromosome 9 by a normal sperm, followed by monosomic rescue to lead to paternal iUPD9. The formation of XXY/XY mosaicism may be due to trisomic rescue, that is, a normal sperm fertilizes a multi-X and uni-9 egg, or a uni-9 egg fertilizes a multi-X sperm, and after trisomic rescue, the redundant X chromosome cannot be completely excreted.

Prenatal diagnosis of UPD is difficult to manage in genetic counseling because of our limited knowledge of the complicated genetic effects of UPD and of imprinted genes. This is the first case report of paternal iUPD9 with mosaic Klinefelter’s syndrome, and no abnormality has been observed during the 3.5-year follow-up. Further observation is required to determine whether the imprinted genes on the chromosomes are pathogenic and whether recessive pathogenetic genes are activated. We will continue to follow this case.

## Data Availability

The datasets used and/or analysed during the current study are available from the corresponding author on reasonable request.

## Abbreviations

aCGH: Array-comparative genomic hybridization
GLIS-3: GLIS family zinc finger 3
GTG: G band Trypsin Giemsa
ICSI: Intracytoplasmic sperm injection
iUPD: isodisomy Uniparental disomy
IVF: In vitro fertilization
PGT-SR: Preimplantation genetic testing for chromosomal structural rearrangements
PKU: Phenylketonuria
SIGMAR1: Sigma non-opioid intracellular receptor 1
SNP: Single nucleotide polymorphism
UPD: Uniparental disomy
